# High-risk Brugada syndrome: factors associated with arrhythmia recurrence and benefits of epicardial ablation in addition to implantable cardioverter defibrillator implantation

**DOI:** 10.1093/europace/euae019

**Published:** 2024-01-22

**Authors:** Vincenzo Santinelli, Giuseppe Ciconte, Francesco Manguso, Luigi Anastasia, Emanuele Micaglio, Zarko Calovic, Gabriele Vicedomini, Beniamino Mazza, Mattia Vecchi, Valerio Mecarocci, Emanuela T Locati, Antonio Boccellino, Gabriele Negro, Antonio Napolano, Luigi Giannelli, Carlo Pappone

**Affiliations:** Arrhythmology Department, IRCCS Policlinico San Donato, Piazza E Malan, 20097 San Donato Milanese, Italy; Arrhythmology Department, IRCCS Policlinico San Donato, Piazza E Malan, 20097 San Donato Milanese, Italy; Arrhythmology Department, IRCCS Policlinico San Donato, Piazza E Malan, 20097 San Donato Milanese, Italy; Arrhythmology Department, IRCCS Policlinico San Donato, Piazza E Malan, 20097 San Donato Milanese, Italy; Arrhythmology Department, IRCCS Policlinico San Donato, Piazza E Malan, 20097 San Donato Milanese, Italy; Arrhythmology Department, IRCCS Policlinico San Donato, Piazza E Malan, 20097 San Donato Milanese, Italy; Arrhythmology Department, IRCCS Policlinico San Donato, Piazza E Malan, 20097 San Donato Milanese, Italy; Arrhythmology Department, IRCCS Policlinico San Donato, Piazza E Malan, 20097 San Donato Milanese, Italy; Arrhythmology Department, IRCCS Policlinico San Donato, Piazza E Malan, 20097 San Donato Milanese, Italy; Arrhythmology Department, IRCCS Policlinico San Donato, Piazza E Malan, 20097 San Donato Milanese, Italy; Arrhythmology Department, IRCCS Policlinico San Donato, Piazza E Malan, 20097 San Donato Milanese, Italy; Arrhythmology Department, IRCCS Policlinico San Donato, Piazza E Malan, 20097 San Donato Milanese, Italy; Arrhythmology Department, IRCCS Policlinico San Donato, Piazza E Malan, 20097 San Donato Milanese, Italy; Arrhythmology Department, IRCCS Policlinico San Donato, Piazza E Malan, 20097 San Donato Milanese, Italy; Arrhythmology Department, IRCCS Policlinico San Donato, Piazza E Malan, 20097 San Donato Milanese, Italy; Arrhythmology Department, IRCCS Policlinico San Donato, Piazza E Malan, 20097 San Donato Milanese, Italy; University Vita-Salute San Raffaele, Via Olgettina 58, 20132 Milan, Italy

**Keywords:** Brugada syndrome, Sudden cardiac death, Syncope, Catheter ablation, Ventricular fibrillation, Outcome

## Abstract

**Aims:**

This study aims to evaluate the prognostic impact of the arrhythmogenic substrate size in symptomatic Brugada syndrome (BrS) as well as to validate the long-term safety and effectiveness of epicardial radiofrequency ablation (RFA) compared with no-RFA group.

**Methods and results:**

In this prospective investigational long-term registry study, 257 selected symptomatic BrS patients with implantable cardioverter defibrillator (ICD) implantation were included. Among them, 206 patients underwent epicardial RFA and were monitored for over 5 years post-ablation (RFA group), while 51 patients received only ICD implantation declining RFA. Primary endpoints included risk factors for ventricular fibrillation (VF) events pre-ablation and freedom from VF events post-ablation. In the RFA group, BrS substrates were identified in the epicardial surface of the right ventricle. During the pre-RFA follow-up period (median 27 months), VF episodes and VF storms were experienced by 53 patients. Independent risk factors included substrate size [hazard ratio (HR), 1.13; 95% confidence interval (CI), 1.08–1.18; *P* < 0.001], aborted cardiac arrest (HR, 2.98; 95% CI, 1.68–5.28; *P* < 0.001), and *SCN5A* variants (HR, 2.22; 95% CI, 1.15–4.27; *P* = 0.017). In the post-RFA follow-up (median 40 months), the RFA group demonstrated superior outcomes compared with no-RFA (*P* < 0.001) without major procedure-related complications.

**Conclusion:**

Our study underscores the role of BrS substrate extent as a crucial prognostic factor for recurrent VF and validates the safety and efficacy of RFA when compared with a no-RFA group. Our findings highlight the importance of ajmaline in guiding epicardial mapping/ablation in symptomatic BrS patients, laying the groundwork for further exploration of non-invasive methods to guide informed clinical decision-making.

## Graphical Abstract

**Graphical Abstract:**
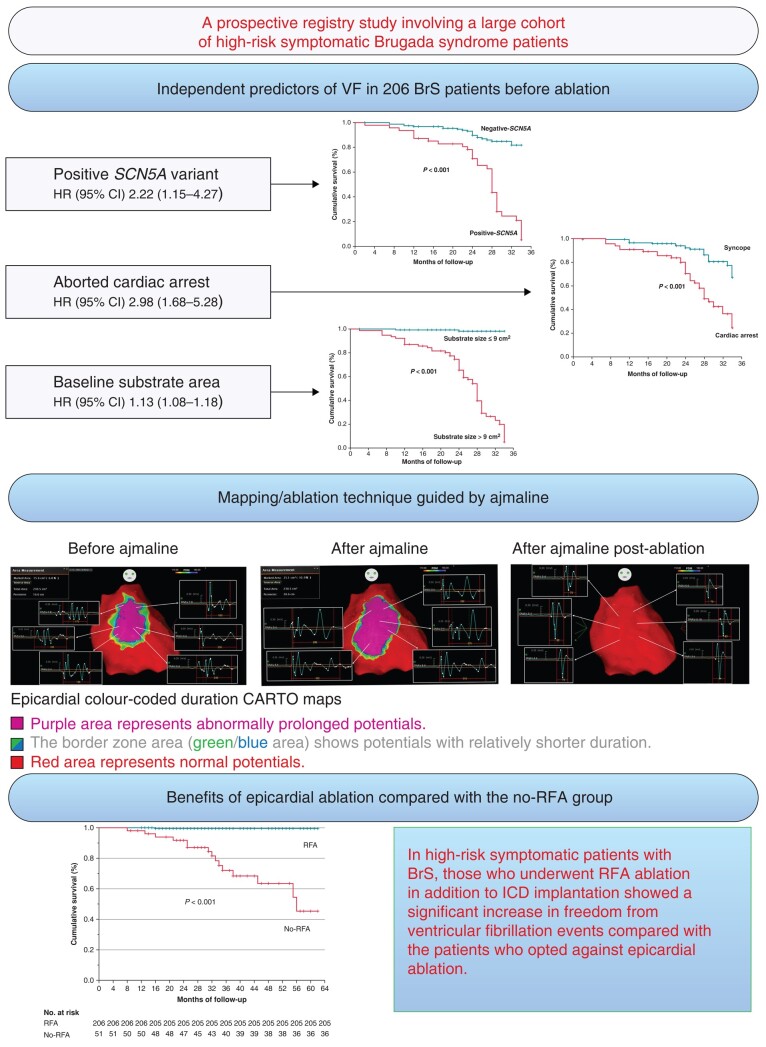
Upper panel: Independent predictors determined by multivariable Cox analysis along with the respective Kaplan–Meier curves. Middle panel: Epicardial maps of the Brugada syndrome substrate before and after ajmaline infusion pre-ablation, as well as after ajmaline infusion post-ablation. Notably, after the ablation, the substrate is eliminated, even in the presence of ajmaline infusion. Lower panel: Long-term benefits of epicardial ablation in comparison with the no-radiofrequency ablation group.

**What’s new?**

- This study underscores the relationship between the extent of arrhythmogenic substrate and long-term clinical outcomes, involving a sizable cohort of symptomatic Brugada syndrome (BrS) patients.
- Extensive substrates located within the right ventricular epicardium provide strong evidence of an increased vulnerability to ventricular fibrillation (VF) storms.
- Guided by ajmaline, substrate mapping and ablation identifies latent or residual BrS substrates, and their complete elimination is associated with consistent post-ablation normalization of the electrocardiogram (ECG) pattern up to 6 years after the procedure.
- The sustained and consistent normalization of the type 1 ECG pattern following a single substrate ablation correlates with excellent outcomes compared with no-radiofrequency ablation (RFA) group.
- Considering these compelling findings, it is advisable to consider adjunctive epicardial ablation guided by ajmaline administration as a safe and more effective strategy to prevent VF events and VF storms, thus reducing the need for multiple ablations.
- By emphasizing the critical role of epicardial ablation in managing BrS, our results establish a solid foundation for future exploration of non-invasive methods to accurately determine the substrate size for clinical decision-making.

## Introduction

Brugada syndrome (BrS) is characterized by symptoms like syncope, cardiac arrest, or sudden death due to ventricular fibrillation (VF) in generally relatively young individuals without structural heart disease. Patients with initial symptoms are at high risk of further VF events if left untreated.^1–3^ Recurrent life-threatening arrhythmias are unpredictable and can emerge during childhood or later stages.

Initially, treatment options included implantable cardioverter defibrillator (ICD) implantation to stop VF and quinidine therapy to prevent recurrent VF, but chronic quinidine often causes intolerable side effects. Ventricular fibrillation recurrences may persist for years, leading to significant psychological issues, such as reactive depression, increased morbidity, and, distressingly, even suicidal thoughts.^4^

Catheter ablation of ventricular ectopic beats triggering VF within the right ventricular (RV) outflow tract (RVOT) endocardium was initially attempted, but its effectiveness was limited.^5^ In 2011, Nademanee *et al*.^6^ first proposed eliminating abnormal electrical potentials located in the RV epicardium in nine symptomatic BrS patients, with reappearance of BrS ECG pattern and VF recurrence just in one patient after ablation. Subsequently, accurate identification and complete elimination of arrhythmogenic substrate areas displaying abnormal electrical potentials induced by sodium channel blockers were proposed.^7–9^ A prior pilot study involving 135 BrS patients showed that ECG pattern normalization was promptly achieved post-procedure and consistently persisted up to the 10-month post-ablation mark without any complications.^8^

Recent advances have improved the treatment for symptomatic BrS patients compared with two decades ago.^10–12^ A new guideline for the management of ventricular arrhythmias and the prevention of sudden cardiac death has been published, serving as an update to the 2015 guideline on this topic.^10,11^ Epicardial substrate ablation [radiofrequency ablation (RFA)] is now recommended as a last-resort option for severely symptomatic BrS patients who haven’t responded to other treatments, including ICD and quinidine.^10^ Additionally, a more recent review focuses on 10 novel key aspects of current guidelines, with an increasing relevance of catheter ablation of ventricular arrhythmias, including but not limited to symptomatic BrS patients.^12^ Despite the increasing role of catheter ablation in patients with BrS, there’s a lack of comprehensive studies validating the long-term safety and efficacy of substrate ablation, which is a complex and novel procedure.^13–15^ Recent studies provide more evidence on RFA’s safety and efficacy in highly symptomatic patients with BrS, but there’s a need for comparative analysis with control groups, systematic substrate mapping/elimination guidance, and randomized trials, as acknowledged by the same authors.^13–15^

## Study aim

In contrast to our previous three pilot studies,^7–9^ this registry-based BrS study, involving a different and carefully selected large cohort of symptomatic BrS patients, aims to investigate the correlation between the extent of BrS substrate and the recurrence of VF events. Furthermore, the study intends to assess and validate the safety of ajmaline-guided substrate mapping and ablation (RFA) and its resulting clinical impact on long-term outcomes, in comparison with a no-RFA group.

## Methods

### Study participants and design

**Figure 1:**
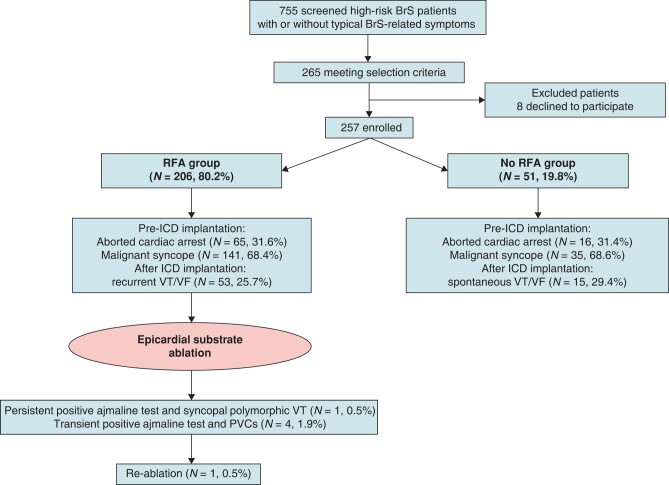
Flowchart of the study’s BrS population: a total of 755 high-risk BrS patients with ICD implantation, included in the registry and with or without typical BrS-related symptoms, were screened. Patients either presented directly to Policlinico San Donato or were referred from various locations across Italy.

*Figure 1* depicts the flowchart of the study population. Consecutive high-risk patients diagnosed with BrS, who had undergone ICD implantation and were presenting with or without typical BrS-related symptoms, were enrolled in the ongoing BrS registry database (NCT03106701) at Policlinico University Hospital San Donato in Italy. The criteria for ICD implantation included documented polymorphic VT or VF, a baseline type 1 ECG pattern in precordial leads ≥ 0.20 mV, borderline type 1 ECG patterns that spontaneously fluctuate, a family history of premature sudden death, and/or inducible sustained VF. None of the participants had previously undergone epicardial mapping or ablation procedures. The registry, initiated in 2015, underwent three pilot studies (2015–2017), which were excluded from the current analysis based on specific criteria. The current cohort, spanning from November 2017 to January 2022, comprises high-risk symptomatic patients who either underwent or did not undergo epicardial mapping and ablation and agreed to follow-up until January 2023.

**Inclusion criteria:**

- Spontaneous or ajmaline-induced type 1 ECG pattern
- Absence of concomitant early repolarization ECG pattern
- Typical BrS-related symptoms [aborted cardiac arrest (ACA) or malignant syncope]
- ICD implantation
- Negative coronary angiography in cardiac arrest survivors
- Diagnostic workup in syncope patients without documented ventricular arrhythmias
- Written informed consent and commitment to epicardial ablation and follow-up at our centre

**Exclusion criteria:**

- Structural heart disease (indicated by imaging and testing)
- Age < 18 years
- Ongoing pregnancy
- Breastfeeding
- Life expectancy ≤ 12 months

A total of 257 eligible symptomatic patients were categorized into RFA and no-RFA groups. Of these, 206 consented to epicardial mapping and ablation, monitored from November 2017 to January 2023 (RFA group). Conversely, 51 opted out, citing concerns about potential complications and uncertainties about long-term benefits of catheter ablation, in line with current guidelines.^1^ However, only a minority declined the procedure.

In the RFA group, the minimum follow-up duration was 12 months, extending beyond 5 years post-ablation. The study protocol was reviewed and approved by the local Institutional Ethics Committee (AR-01-2015), and mapping and ablation procedures were conducted at Policlinico San Donato, Italy, in accordance with the Declaration of Helsinki. Further information on the BrS registry design and population is reported in the Supplementary material online.

### Genetic testing

All participants underwent genetic testing with genomic DNA extracted from peripheral blood and analysed by next-generation sequencing (NGS) technique, as previously described.^16^ More details on genetic testing in both groups are provided in the Supplementary material online, *Table S1*.

### Implantable cardioverter defibrillator implantation

Information on ICD implant can be available in the Supplementary material online.

### Substrate mapping

Electrophysiological study, substrate mapping, and ablation were performed as previously described in detail.^8,9^ Under general anaesthesia, a combined epi- and endocardial mapping procedure was performed in the RFA group. Briefly, electroanatomical mapping was performed with CARTO 3 (Biosense Webster). The epicardial mapping of the arrhythmogenic substrate of the RV and LV epicardium was performed during sinus rhythm using a dedicated high-density mapping catheter (DECANAV catheter, Biosense Webster) to define the duration, location, and size of the abnormal substrate before and after ajmaline infusion (up to 1 mg/kg in 5 min). Abnormal prolonged potentials were identified if they met at least one of the following characteristics: (i) a wide duration (>110 ms) with fragmented component (>3 distinct peaks), (ii) distinct and delayed component exceeding the end of the QRS complex, or (iii) discrete double activity.^9^ The substrate extent was calculated as the percentage of the total RVOT and/or anterior RV wall area. Radiofrequency ablation was systematically performed during sinus rhythm at the epicardial sites where ajmaline infusion had defined abnormal areas, as previously reported.^8^ The RFA procedure was continued until electrogram abnormalities on the epicardial electroanatomic maps were eliminated, and until the type 1 BrS pattern was no longer inducible, even after an ajmaline challenge. Further information on the substrate mapping and ablation is available in the Supplementary material online.

### Follow-up evaluations

Patients underwent systematic follow-up by remote monitoring after ICD implantation and were scheduled for in-person follow-up appointments at 1, 3, 6, and 12 months during the first year, followed by appointments every 6 months up to 24 months, and subsequently annually, with the most recent contact recorded in January 2023.

All participants were monitored using standardized ICD remote monitoring to detect any potential recurrence of arrhythmic events. After RFA, clinic visits and ajmaline tests were systematically conducted according to the same schedule as after ICD implantation.

### Definitions

Malignant syncope: When documentation of life-threatening ventricular arrhythmia was lacking, we conducted a systematic diagnostic workup. This workup involved assessing clinical characteristics, patient history, prodromes, triggers, syncope duration, medication history, family history of syncope or sudden death, genetic testing, neurologic visit with EEG, VF inducibility, and programmed electrical stimulation to exclude supraventricular arrhythmia including atrial fibrillation as cause if syncope. Additional data from various diagnostic tests, including exercise and tilt testing, were collected. A three-member expert committee independently assessed these data to make a final diagnosis of suspected arrhythmic syncope for inclusion in the study.

VF events: Appropriate ICD shocks due to spontaneous sustained polymorphic ventricular tachycardia (VT) and/or VF or VF storms requiring ICD intervention, which included ICD shock or anti-tachycardia pacing (ATP).

Family history of sudden cardiac death: Defined as a sudden unexpected death at or before the age of 45 years in a first- or second-degree family member.

Young BrS patients: Patients aged 30 years or younger at the time of diagnosis.

Electric or VF storms: Occurrence of three or more separate episodes of ventricular arrhythmia requiring ICD therapy within a 24-h period.

### Primary endpoints

The primary endpoints of this study encompassed the identification of risk factors for VF events before ablation and an assessment of the long-term benefits of epicardial ablation in reducing VF recurrence when compared with the no-RFA group.

### Secondary endpoints

The secondary endpoints included the normalization of the BrS ECG pattern, both during and after the procedure, up to the last pre-specified follow-up, as well as the evaluation of any complications.

### Statistical analysis

Continuous variables are either expressed as median (interquartile range) or mean (standard deviation) depending on their distribution. Categorical variables are expressed as frequency and percentage. For comparing continuous variables, the Mann–Whitney *U* test, the Wilcoxon signed tank test, or the unpaired *t*-test were applied. Categorical variables were compared using the Pearson χ^2^. Cumulative survival was evaluated using the Kaplan–Meier (KM) method, with the time of ICD implantation as the baseline and the time to first event as the endpoint. Within the RFA group, differences between subgroups (e.g. ACA vs. syncope, patients with/without *SCN5A* variants, patients with substrate size ≤ 9 vs. >9 cm^2^, male vs. female patients) were assessed using the log-rank test. Data were censored in cases of ablation, study completion, death, or loss to follow-up before an event or ablation. In the RFA group, differences in outcomes pre- and post-RFA were determined using the log-rank test. To compare outcomes between the RFA and no-RFA groups, we employed the KM method. For the RFA group, the starting point was the time of RFA, and for the no-RFA, it was the time they declined ablation. The time to the first event served as the endpoint for both groups. Differences between the groups were evaluated using the log-rank test. For identifying factors associated with events before RFA, Cox proportional hazards model was utilized. Univariable and multivariable models with backward-stepwise selection and distance tests based on the Wald probability statistic were employed. Covariates included age, sex (male/female = 0/1), baseline BrS ECG pattern (others/type 1 = 0/1), *SCN5A* variant (negative/positive = 0/1), clinical presentation (syncope/aborted cardiac arrest = 0/1), baseline substrate area before ajmaline (as a continuous value, per cm^2^ increase), and inducibility of VT/VF (no/yes = 0/1). Analyses were performed both unadjusted and adjusted for covariates (i.e. analysis adjusted for confounders). The proportional hazards assumption was verified using log–log plots, showing no significant discrepancies. A receiver operating characteristic (ROC) curve was generated to assess the predictive performance of substrate size in VF prediction, calculating the area under the curve. The substrate size threshold was determined based on the Youden index, with confidence intervals (CIs) for the cut-off point determined using a bootstrap method (2000 permutations in RStudio version 2022.07.1, Posit, Boston, Massachusetts). The ROC curve was generated using StatsDirect statistical software (version 3.3.6, StatsDirect, Ltd., Altrincham, UK). Two-sided *P* values of <0.05 were considered significant. SPSS Statistics for Windows version 27.0.1.0 software (IBM Corp., Armonk, New York) was used for statistical analysis. Stata/BE 17.0 was used to test the proportionality to hazards assumption.

## Results

### Patient characteristics

The study participant flowchart is depicted in *Figure 1*. Out of 755 high-risk patients with BrS screened and having an ICD, 265 were selected, and 8 patients who experienced ACA declined participation (note that these patients are confirmed alive based on local contact). Among the 257 enrolled patients, 65 with ACA and 141 with malignant syncope underwent epicardial ablation (RFA group, 206 patients), while 16 with ACA and 35 with syncope received only an ICD implantation and were subsequently monitored (no-RFA group, 51 patients). No significant differences were observed when comparing the characteristics between these two groups (*Table 1*).

**Table 1:** Comparison of characteristics between the RFA and no-RFA groups

Characteristics	RFA Group (*n* = 206)	No-RFA Group (*n* = 51)	*P*-value
Male sex—*n* (%)	143 (69.4)	34 (66.7)	0.704
Age at diagnosis—years, mean ± SD	40.5 ± 11.6	39.1 ± 8.3	0.340
Min–max range	18–68	18–55	
Pre-ICD type 1 ECG pattern—*n* (%)	43 (20.9)	11 (21.6)	0.913
Family history of SCD—*n* (%)	71 (34.5)	18 (35.3)	0.911
Positive *SCN5A* variant—*n* (%)	47 (22.8)	12 (23.5)	0.914
Inducible VT/VF	114 (55.3)	29 (56.9)	0.845
Substrate size—cm^2^	5.8 (2.1–12.0)	–	–
Min–max range	0–56.6		
Substrate size after ajmaline—cm^2^	16.6 (12.6–24.5)	–	–
Min–max range	2.3–64.2		
^a^ICD follow-up—months	27 (22–29)	26 (23–28)	0.530
Min–max range	2–34	3–34	
^b^Outcome follow-up—months	40 (24–58)	33 (25–46)	0.063
Min–max range	12–62	8–62	

Data are medians and interquartile ranges except when indicated.

BrS, Brugada syndrome; ECG, electrocardiogram; IQR, interquartile range; RFA, radiofrequency ablation; SD, standard deviation; SCD, sudden cardiac death; VT/VF, ventricular tachycardia/fibrillation.

^a^For the RFA group, ‘ICD follow-up’ refers to the period between ICD implantation and RFA ablation. In the no-RFA group, ‘ICD follow-up’ denotes the time span between ICD implantation and the decision to refuse ablation.

^b^For the RFA group, ‘outcome follow-up’ refers to the time span between RFA ablation and the most recent follow-up contact. In the no-RFA group, ‘outcome follow-up’ represents the period from when the ablation was declined to the latest follow-up contact. This ensures a comparable starting point for both groups by excluding the interval between ICD implantation and the decision to decline the procedure.

In the RFA group, the age distribution is illustrated in Supplementary material online, *Figure S1*. Among ACA patients in the RFA group, higher rates of VT/VF inducibility were observed compared with the syncope group, whereas in the no-RFA group, no significant differences were noted between ACA and syncope patients (see Supplementary material online, *Table S2*). Despite the RFA group being predominantly male, no significant differences in baseline characteristic were observed based on sex (see Supplementary material online, *Table S3*). The RFA group included 52 patients aged 30 years or younger, and no significant differences in baseline characteristics were observed between these two subgroups (see Supplementary material online, *Table S4*). Further details on both groups are provided in the Supplementary material online.

### Ventricular fibrillation burden after implantable cardioverter defibrillator implantation

Over a median follow-up period of 27 months after ICD implantation before ablation, 53 out of 206 patients (25.7%) reported experiencing dizziness or pre-syncope accompanied by palpitations (*Figure 1* and *Table 2*). These symptoms were attributed to VT/VF or VF storms, which were substantiated by ICD data, leading to single or multiple appropriate ICD shocks. Among these patients, 31 were survivors of ACA, while 22 were syncope patients without documented VF either during the syncope episode or later before ICD implantation (see Supplementary material online, *Table S5*). Notably, 13 patients (11 in the ACA group and 2 in the syncope group) exhibited a type 1 Brugada pattern > 2 mV during post-ICD implantation follow-up visits, even though their first ECGs exhibited borderline coved ST-elevation < 0.2 mV. In total, 109 appropriate ICD shocks were delivered, with 26 patients receiving a single shock and 27 patients receiving multiple shocks (ranging from 2 to 10) for VF (33 patients) or VF storms (20 patients, 16 with ACA), all while on chronic hydroquinidine therapy. Short-term administration of isoproterenol was needed for patients with VF storms. In 10 patients, following ICD discharges, 12-lead ECGs exhibited frequent PVCs characterized by left bundle branch block (LBBB) morphology and an inferior axis configuration, which triggered VF. In comparison with those without spontaneous VF events, patients with VF events were more frequently individuals who survived ACA, more often had positive genetic testing, demonstrated VT/VF inducibility, and exhibited a baseline type 1 ECG pattern (*Table 2*).

**Table 2:** Characteristics of the RFA group experiencing VF events before ablation

Characteristics	Events		*P*-value
	Yes (*n* = 53)	No (*n* = 153)	
Male sex—*n* (%)	37 (69.8)	106 (69.3)	0.942
Age at diagnosis—years, means ± SD	41.3 ± 10.9	40.2 ± 11.9	0.532
Min–max range	20–63	18–68	
Spontaneous type 1 ECG pattern—*n* (%)	17 (32.1)	26 (17.0)	0.020
Family history of SCD—*n* (%)	12 (22.6)	59 (38.6)	0.036
Positive *SCN5A* variant—*n* (%)	33 (62.3)	14 (9.2)	<0.001
Inducible VT/VF—*n* (%)	39 (73.6)	75 (49.0)	0.002
Prior cardiac arrest—no. (%)	31 (58.5)	34 (22.2)	<0.001
Median baseline substrate size (IQR)—cm^2^	13.6 (12.0–15.1)	3.9 (0.9–7.0)	<0.001
Min–max range	9–56.6	0–22.1	
Median substrate size after ajmaline (IQR)—cm^2^	25.0 (16.5–28.7)	15.4 (12.1–21.3)	<0.001
Min–max range	6.4–64.2	2.3–36.6	

ECG, electrocardiogram; IQR, interquartile range; RFA, radiofrequency ablation; SD, standard deviation; SCD, sudden cardiac death; VT/VF, ventricular tachycardia/fibrillation.

### Relationship between ventricular fibrillation burden and Brugada syndrome substrate extent

**Figure 2:**
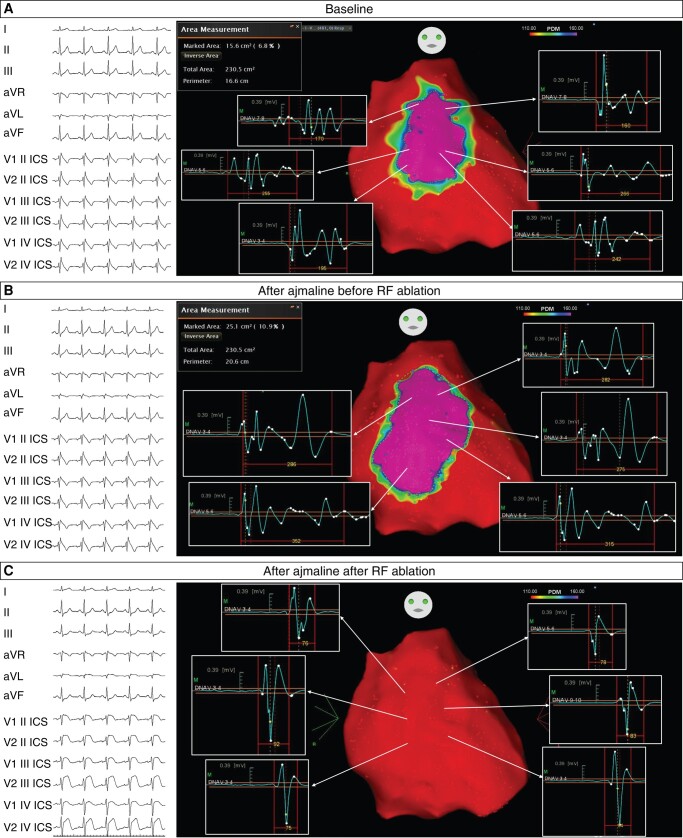
The figure shows three epicardial substrate maps of a 22-year-old patient presenting with aborted cardiac arrest, spontaneous type 1 ECG pattern, and *SCN5A* gene variant. Before ablation, the patient had VF storms requiring multiple ICD shocks. A baseline large (15.6 cm^2^) arrhythmogenic area was identified on the anterior RVOT epicardium, characterized by prolonged, fragmented low-voltage potentials (*A*). After ajmaline (*B*), the abnormal area increased to 25.1 cm^2^, and this finding was associated with a concomitant increase of the J-point elevation in precordial leads recorded from the second to the fourth intercostal space. Note that epicardial ablation of the targeted area resulted in non-inducibility of the type 1 BrS ECG pattern under ajmaline infusion (*C*).

Overall, substrate mapping revealed that the median baseline substrate size was 5.8 cm^2^, which significantly increased to 16.6 cm^2^ following ajmaline administration (*P* < 0.001; *Table 1*). Patients experiencing VF events exhibited larger substrates both at baseline and after ajmaline administration compared with those without such events (*Table 2*). Among patients with VF events before ablation, those who survived cardiac arrest (*n* = 31) displayed wider substrates after ajmaline administration compared with syncope patients (*P* < 0.001; Supplementary material online, *Table S5*). Furthermore, patients who experienced VF storms within the group with events exhibited broader baseline substrates, characterized by fragmented, delayed, and low-voltage potentials (*P* < 0.001; *Table 3*). Suspicious BrS ECG patterns were detected after ajmaline reinfusion in 84 patients, requiring additional RF applications during the same hospitalization session to eliminate the remaining abnormal potentials. Complete substrate ablation, disappearance of the BrS ECG pattern, and non-inducibility of VT/VF were successfully achieved in all patients upon hospital discharge (*Figure 2*). Prophylactic antibiotics were administered for 7 days, along with the appropriate use of steroidal and/or non-steroidal anti-inflammatory drugs as required.

**Table 3:** Characteristics of the RFA group experiencing VF storms before ablation

Characteristic	Events		*P*-value
	VF storms (*n* = 20)	VF (*n* = 33)	
Male sex—no. (%)	14 (70)	23 (69.6)	0.981
Age at diagnosis—years	44.9 ± 11.9	39.2 ± 9.8	0.067
Min–max	22–63	20–59	
Spontaneous type 1 ECG pattern—*n* (%)	7 (35)	10 (30.3)	0.723
Family history of SCD—*n* (%)	5 (25)	7 (21.2)	0.748
Positive *SCN5A*—*n* (%)	12 (60)	21 (63.6)	0.791
Inducible VT/VF—no. (%)	17 (85)	22 (66.7)	0.142
Median baseline substrate size (IQR)—cm^2^	15.1 (14.4–16.4)	12.4 (11.4–14)	<0.001
Min–max	9.8–56.6	9–23.9	
Median substrate size after ajmaline (IQR)—cm^2^	25.7 (21.1–29.0)	22.1 (14.6–28.7)	0.071
Min–max	13–64.2	6.4–34.7	

ECG, electrocardiogram; IQR, interquartile range; SCD, sudden cardiac death; VT/VF, ventricular tachycardia/fibrillation; RFA, radiofrequency ablation.

### Risk factors associated with ventricular fibrillation burden before ablation

**Figure 3:**
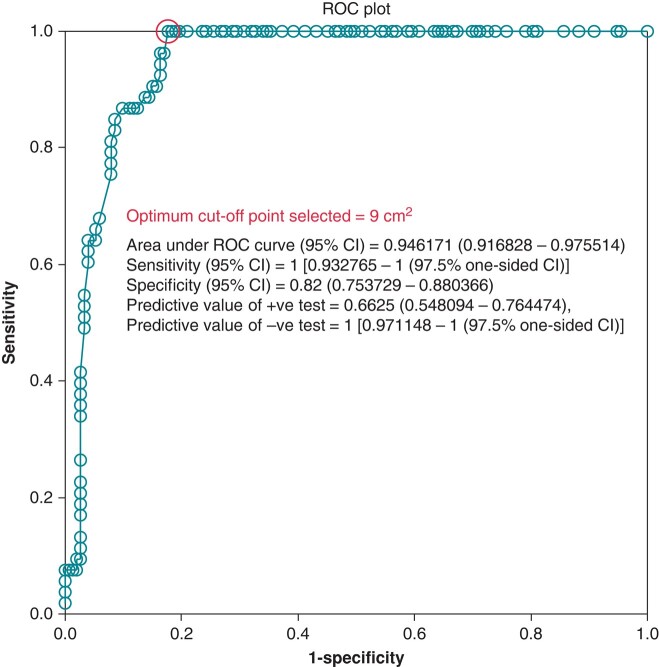
The ROC curve analysis identified an optimal cut-off point of 9 cm^2^ for detecting symptomatic patients at risk of recurrent VF after the initial symptom.

**Figure 4:**
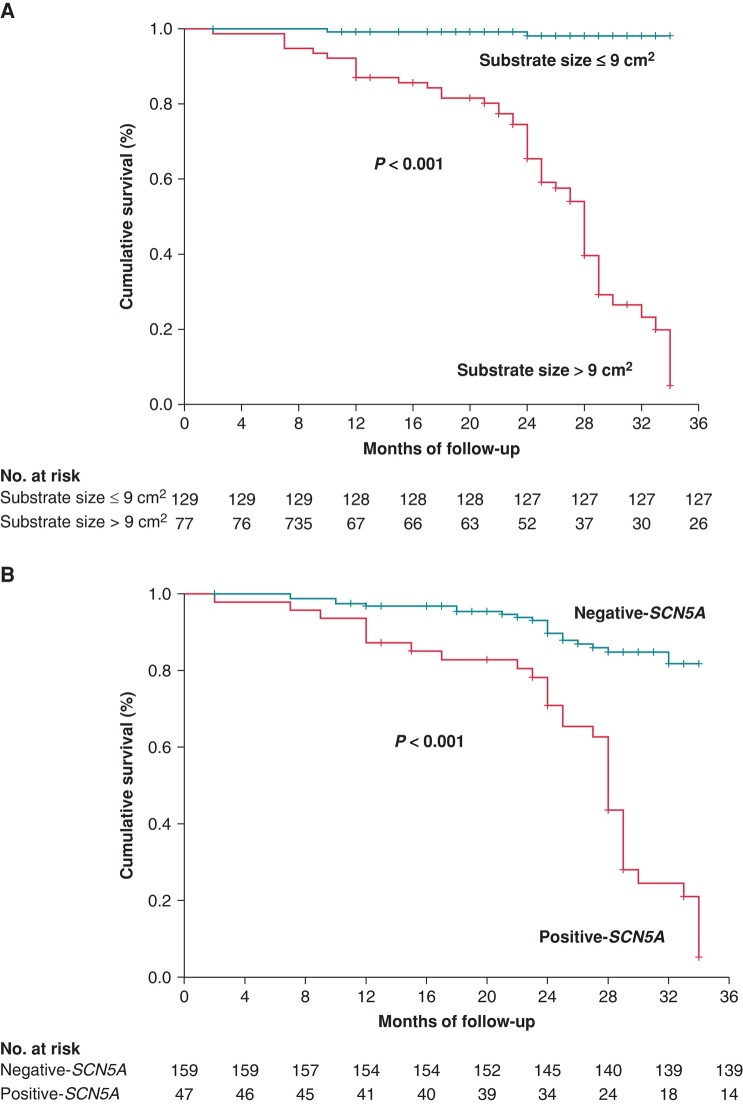
A: cumulative survival shows that patients with substrates above 9 cm^2^ (*A*) or those with *SCN5A* variants (*B*) were more likely to develop VF events after ICD implantation.

In the multivariable adjusted analysis, baseline substrate size, a history of ACA, and *SCN5A* variants were identified as significant risk factors for episodes of VF burden (*Table 4*). The ROC curves demonstrated a cut-off point of 9 cm^2^ (Youden index = 0.823; 95% CI: 8.36–11.1 cm^2^) that predicted VF events (*Figure 3*). The KM curves revealed that patients with substrates > 9 cm^2^ (*Figure 4A*) or patients with *SCN5A* variants (*Figure 4B*) were more likely to experience VF events before ablation. No significant differences related to sex were observed in the survival curves (see Supplementary material online, *Figure S2*).

**Table 4:** Predictors of VF events using the Cox proportional hazard models

Variables^a^	Univariable				Multivariable			
	Regression coefficient	*P*-value	HR	95% CI	Regression coefficient	*P*-value	HR	95% CI
Age	0.005	0.684	1.01	0.9–1.03	–	–	–	–
Sex	−0.118	0.694	0.89	0.49–1.60	–	–	–	–
Spontaneous type 1 ECG pattern	0.525	0.076	1.69	0.95–3.01	–	–	–	–
Inducible VT/VF	0.747	0.017	2.11	1.15–3.90	–	–	–	–
Positive *SCN5A* variant	1.787	<0.001	5.97	3.43–10.42	0.796	0.017	2.22	1.15–4.27
Aborted cardiac arrest	1.342	<0.001	3.83	2.21–6.63	1.092	<0.001	2.98	1.68–5.28
Baseline substrate area	0.152	<0.001	1.16	1.12–1.21	0.120	<0.001	1.13	1.08–1.18

CI, confidence interval; ECG, electrocardiogram; HR, hazard ratio; VT/VF, ventricular tachycardia/fibrillation.

^a^All variables were dichotomous (0/1), except baseline substrate area that was used as continuous variable with 1 cm^2^ steps.

### Safety of radiofrequency ablation and outcome differences between radiofrequency ablation and no-radiofrequency ablation groups

At the conclusion of the study, all participants remained alive, and no instances of lost to follow-up were recorded in either group throughout the study period.

#### Outcomes after RFA

Within the RFA group, 79 patients were initially prescribed chronic quinidine therapy; however, due to intolerable side effects, 53 patients had to discontinue this treatment. The pre-RFA survival curves according to clinical presentation showed higher event rates in cardiac arrest survivors than in syncope patients (see Supplementary material online, *Figure S3A*). After ablation, during the observation period, only one arrhythmic event was recorded in the ACA group, without the use of antiarrhythmic drugs (see Supplementary material online, *Figure S3B*). Moreover, the ECG pattern underwent normalization immediately after the ablation procedure, leading to the disappearance of the type 1 BrS pattern in all patients. This normalization persisted during the post-RFA follow-up for all patients, with the exception of one individual during a median follow-up of 40 months. This unique case pertained to a 41-year-old male with a history of ACA, a baseline type 1 ECG pattern, inducible VT/VF, and a variant in the *SCN5A* gene. This particular patient required a repeat ablation due to sustained VT, which was effectively managed using single ATP therapy. Over the post-RFA follow-up period, this patient exhibited a positive ajmaline test that turned negative only after re-ablation and the elimination of residual substrates. During the initial RFA procedure, he had encountered six appropriate ICD shocks for VF storms, despite receiving quinidine therapy. Initial mapping revealed a substantial substrate area (measuring 56.6 cm^2^) extending from RVOT epicardium to the anterior wall of the right ventricle. Upon ajmaline infusion, the patient experienced sustained VF and cardiac arrest, promptly managed using an ICD shock, which subsequently precluded further ajmaline testing to ensure comprehensive substrate elimination. Subsequent remapping indicated the presence of 9.2 cm^2^ of residual abnormal areas, as unveiled by ajmaline infusion. Additionally, four patients initially presenting with syncope and documented episodes of malignant VA showcased extensive substrates (>9 cm^2^) covering a substantial portion of the anterior RVOT epicardium. Although these patients exhibited frequently isolated PVCs characterized by a LBBB and inferior axis, these PVCs failed to induce VA post-RFA. After the initial ablation, these four patients demonstrated a transiently positive ajmaline test only during the first follow-up, with subsequent tests returning negative by the last follow-up. Echocardiography performed during each follow-up visit consistently revealed normal results.

### Outcomes in the no-radiofrequency ablation group

In the no-RFA group, following ICD implantation and prior to declining ablation, 12 patients experienced ICD-documented self-terminating episodes of VF over a median follow-up of 26 months. Among these 12 patients, 7 had a history of ACA, while 5 presented with syncope without any documented ventricular arrhythmias before their ICD implantation. Additionally, six of these patients (four of whom had ACA and two with syncope) who initially lacked a first baseline type 1 pattern later demonstrated a stable type 1 Brugada ECG pattern during their post-ICD implantation follow-up visits.

During a median follow-up period of 33 months after opting out of RFA, 15 patients, including the first aforementioned 12 patients, along with 3 additional patients, experienced ICD-recorded VF events (*Figure 1* and *Table 5*). In response to VF episodes, these 15 patients (8 ACA and 7 syncope patients) received single or multiple ICD shocks for VF termination, despite chronic quinidine therapy, with dosages reaching up to 900 mg per day in 10 patients. Seven patients were administered a single appropriate shock, while eight patients received between two and eight shocks each. However, none of these patients experienced VF storms during the follow-up period.

**Table 5:** Characteristics of the no-RFA group who experienced VF events after opting out of ablation

Characteristics	No-RFA group		*P*-value
	Events		
	Yes (*n* = 15)	No (*n* = 36)	
Male sex—*n* (%)	10 (66.7)	24 (66.7)	1.000
Age at diagnosis—years, means ± SD	38.1 ± 9.0	39.5 ± 8.1	0.590
Min–max range	18–53	19–55	
Spontaneous type 1 ECG pattern—*n* (%)	2 (13.3)	9 (25)	0.472
Family history of SCD—*n* (%)	4 (26.7)	14 (38.9)	0.405
Positive *SCN5A* variant—*n* (%)	5 (33.3)	7 (19.4)	0.302
Inducible VT/VF—*n* (%)	11 (73.3)	18 (50)	0.125
Prior cardiac arrest—no. (%)	8 (53.3)	8 (22.2)	0.047

ECG, electrocardiogram; RFA, radiofrequency ablation; SD, standard deviation; SCD, sudden cardiac death; VT/VF, ventricular tachycardia/fibrillation.

### Comparison between radiofrequency ablation and no-radiofrequency ablation outcomes

**Figure 5:**
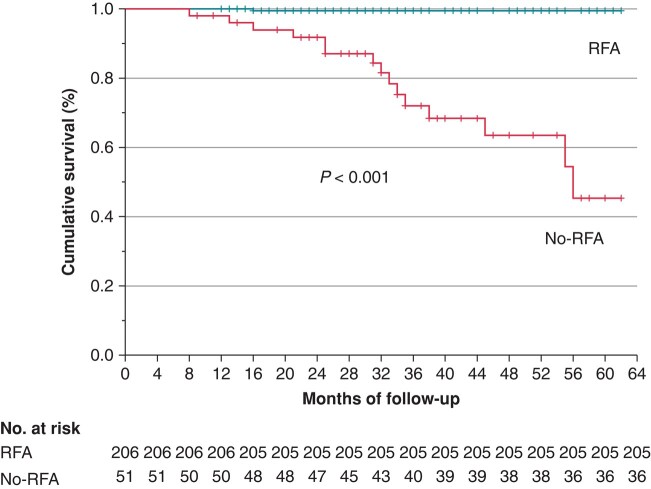
The figure shows that the RFA group experienced a drastic reduction in appropriate ICD shocks after ablation compared with the no-RFA group. The reported KM cumulative survival curves were initiated from a common point, corresponding to the time of ablation for the RFA group and the time of refusal ablation for the no-RFA group. Patients with events ‘after declining ablation’ encompass those that recurred in the same 12 patients who had VF events before refusing ablation, along with 3 additional patients, totalling 15 patients.

Freedom from VF events after ablation displayed a significant increase compared with the no-RFA outcomes (*P* < 0.001; *Figure 5*).

## Discussion

Identifying risk factors for recurrent VF episodes is crucial for timely intervention in symptomatic BrS patients. In our non-randomized interventional study, complemented with a no-RFA group, we examined a cohort of 257 symptomatic BrS patients with prior ICD implantation who initially presented with either cardiac arrest or malignant syncope. We categorized them into two groups: those who underwent epicardial ablation and those who did not. Our study encompasses a wide range of data, including clinical, ECG, genetic and electrophysiological characteristics. We also considered ICD arrhythmia documentation, mapping/ablation data, and pre- and post-RFA follow-ups. Remarkably, we retained all patients throughout this comprehensive long-term follow-up after both ICD implantation and RFA in both groups. From our findings, a pivotal observation from the RFA group was the relevant impact of substrate extent area on long-term outcomes prior to ablation. The optimal substrate size threshold was pinpointed at 9 cm^2^. Notably, many patients with syncope showed a borderline coved ECG pattern <0.2 mV and thus were not considered as type 1 BrS pattern. This phenomenon was primarily influenced by the extent of the underlying substrate, suggesting the inclusion of a potentially lower-risk patient population. The inability of the baseline type 1 BrS ECG pattern to independently predict outcomes in our study may be attributed to these potentially consistent ECG pattern changes, a phenomenon well documented due to the transient nature of the pattern.^17^ In our study, we noticed that, regardless of clinical presentation, a significant number of patients who experienced VF events and lacked a type 1 pattern at baseline transitioned to the typical pattern during follow-up after ICD implantation. While the SABRUS survey reported a higher baseline type 1 ECG pattern rate (69%) in high-risk BrS patients,^18^ larger registry studies like the FINGER study indicated lower rates of type 1 ECG pattern on a single baseline ECG in similar BrS populations.^1^ The FINGER study emphasized that risk stratification primarily relied on a single baseline ECG, underscoring the importance of recognizing the well-documented ST-segment elevation fluctuations over time as a characteristic feature of the syndrome.^17^ Similarly, the dynamic character of VF inducibility, primarily influenced by substrate extent,^9^ did not maintain its position as independent predictor of VF events. These findings are consistent with the FINGER study’s conclusions, where less than half of cardiac arrest survivors or syncope patients showed VT/VF inducibility and/or a diagnostic BrS ECG pattern at baseline.^1^ In contrast, within our analysis, an *SCN5A* gene variant emerged as a standout independent predictor. This finding aligns with prior studies^1,16^ and highlights the necessity for genetic testing in BrS suspects, regardless of the absence of a baseline type1 ECG pattern on a single ECG.

One revelation was that, regardless of their initial clinical presentation, symptomatic patients with extensive arrhythmogenic substrate areas were more prone to recurrent VF storms. Although the post-ICD implantation follow-up period in our study was relatively short, it’s noteworthy that within this time frame, we observed a clear trend. Patients with wider substrates experienced more VF recurrences shortly after ICD implantation, typically within 1–2 years, suggesting that a larger substrate area indeed increases the risk of recurrent VF. Conversely, patients with smaller substrates appeared to be at lower risk. While longer follow-up may provide more comprehensive insights, these initial findings are indeed significant and indicative of the association between substrate size and recurrent VF risk.

Hence, accurate identification and thorough elimination of expansive arrhythmogenic areas are pivotal in drastically reducing or even eliminating VF recurrences including but not limited to VF storms. Solely banking on severe clinical presentation and baseline ECG pattern may be challenging, given that even cardiac arrest survivors might have a prior, undefined syncope history.^2,18,19^ A multi-dimensional diagnostic strategy is indeed indispensable to select unexplained syncope cases for ICD implantation and potential substrate ablation in selected cases. The need of systematic diagnostic evaluations is emphasized by the observation that 22 syncope patients enrolled in our study and without any prior documentation of VF had ICD-documented VF. Lastly, while electroanatomical mapping is currently the gold standard for determining substrate size, our findings suggest further exploring non-invasive techniques.^20–22^

### Efficacy of epicardial ablation compared with no-radiofrequency ablation group

Positive outcomes from epicardial substrate ablation in high-risk symptomatic BrS populations, as documented in previous studies, have been marred by several limitations.^9,13–15,23,24^ These include varying learning curves across centres, diverse sample sizes, treatment heterogeneity, a range of mapping/ablation protocols potentially impacting substrate identification and ablation efficiency, variable follow-up durations, different data collection methodologies, and notably, a lack of control groups. We aimed to address these constraints by adopting a uniform mapping/ablation technique, utilizing ajmaline infusion, consistent long-term follow-ups, and introducing a no-RFA group for a thorough analysis. We aspired to fully eliminate arrhythmogenic substrate areas, ensuring a stable and lasting ECG pattern normalization and excellent outcomes, even in the absence of antiarrhythmic drug therapy.

In our study, during the procedure, approximately half of the RFA group displayed a type 1 ECG pattern and residual abnormal potentials post-ajmaline infusion. This necessitated further RF applications to solidify ECG pattern normalization after reinfusion. Using this approach during the same hospitalization, we were able to consistently normalize the ECG pattern by the procedure’s conclusion, leading to a marked reduction in VF events in the RFA group compared with the pre-RFA period. More importantly, the rate of freedom from recurrent VF events was notably higher compared with the no-RFA group.

A particularly compelling case involved a patient with prior ACA and substantial substrates who, despite quinidine therapy, faced VF storms. In this case, incomplete substrate removal was evident from the reappearance of the typical Brugada ECG pattern during post-RFA follow-up following the ajmaline test, a fact corroborated by remapping. Among the syncope subgroup, four patients displayed an intermittent positive ajmaline test post-RFA, which naturally normalized by the last follow-up. These observations align with our earlier pilot study involving 135 patients (excluded from this study),^8^ wherein swift ECG pattern normalization post-procedure was consistent at the 10-month post-mark.

In essence, our study demonstrates significant long-term efficacy and safety with just one ablation procedure, eliminating the need for antiarrhythmic drugs and/or repeated procedures. These positive outcomes both validate and expand upon a recent study of 159 symptomatic BrS patients undergoing epicardial ablation.^13^ Of these, 31 faced VF recurrences post-ablation, leading 25 patients to opt for a second ablation due to incomplete substrate removal, while 6 refrained. Our experience emphasizes the observation that lingering or dormant arrhythmogenic substrates can significantly contribute to frequent post-RFA VF recurrences, especially when ajmaline isn’t used. These insights underscore the importance of a more comprehensive mapping and ablation strategy to reduce more effectively VF recurrences after the index ablation procedure, minimizing the need for multiple epicardial ablations. While these procedures are safe, they are complex, prolonged, and require general anaesthesia.

### Safety of epicardial ablation

Despite its complex and invasive nature necessitating general anaesthesia, this study demonstrates a favourable safety profile for epicardial ablation. Major complications were rare, consistent with previous studies from experienced centres,^5–9,13–15,24^ but widespread adoption of this complex procedure could carry certain risks, and selection of patients is indeed crucial. Notably, the safety of the ajmaline test, especially the initial test with the substrate remaining intact, holds paramount significance. In our study, sustained VF was observed in just one ACA patient (0.5%) with extensive substrates during drug infusion. Moreover, no abnormal echocardiographic findings were noted post-procedure. Complete substrate elimination is crucial to minimize the need for repeat epicardial ablations.

### Clinical implications

In this study, the incorporation of epicardial ablation, in conjunction with ICD implantation, is indeed a safe and validated strategy for highly symptomatic BrS patients. Our innovative methodology consistently curtails near-fatal or fatal arrhythmias, including VF episodes and/or VF storms, and, more importantly, multiple ICD discharges, eliminating the need for chronic drug therapy and/or repeated procedures, thus enhancing the long-term prognosis for this high-risk group. However, the accurate selection of patients is indeed crucial for an early intervention while minimizing widespread adoption of this procedure.

### Limitations

While our study provides valuable insights, it does have limitations. Randomized studies are a valuable next step after extensive observational studies like ours and the BRAVO study, to ensure the generalizability of the findings and establish epicardial ablation as a novel and viable alternative to ICD implantation.

Additionally, the availability of ajmaline, the substrate identification drug employed in our study, might be restricted in certain regions, potentially affecting the generalizability of our findings.

## Conclusion

Our study underscores, for the first time, the prognostic impact of the extent of BrS substrate in symptomatic BrS patients. Epicardial ablation, when combined with ICD implantation, offers an effective and validated approach to improving outcomes compared with the no-RFA group. Our findings advocate for accurate identification and complete elimination of all arrhythmogenic abnormal potentials to prevent multiple, often refractory VF recurrences or VF storms, and the need for repeated ablations. Our data, in turn, can stimulate further exploration of non-invasive methods to guide critical decisions in the clinical management of symptomatic BrS.

## Supplementary Material

euae019_Supplementary_Data

## Data Availability

The data that support the findings of this study are available from the authors upon a reasonable request.
