# Supplementary material for: High-risk Brugada syndrome: factors associated with arrhythmia recurrence and benefits of epicardial ablation in addition to implantable cardioverter defibrillator implantation
Source: Europace. 2024 Jan 22;26(1):euae019. doi: 10.1093/europace/euae019 (PMC10824473; doi:10.1093/europace/euae019)
Supplement: euae019_Supplementary_Data [file euae019_supplementary_data.docx]

**Supplementary Material**

**High-risk Brugada Syndrome: Factors Associated with Arrhythmia Recurrence and Benefits of Epicardial Ablation in Addition to ICD implantation**

*Vincenzo Santinelli, et al.*

Table of Contents

[**SUPPLEMENTARY METHODS** 3](#_Toc153647952)

[***Study design*** 3](#_Toc153647953)

[***BrS Registry data*** 3](#_Toc153647954)

[***ICD Implantation and follow-up*** 4](#_Toc153647955)

[***Genetic testing*** 5](#_Toc153647956)

[***Abnormal substrate mapping*** 6](#_Toc153647957)

[***Substrate ablation*** 7](#_Toc153647958)

[**SUPPLEMENTARY RESULTS** 7](#_Toc153647959)

[**RFA Group (206 patients)** 7](#_Toc153647960)

[**No-RFA group (51 patients)** 8](#_Toc153647961)

[**ICD complications** 9](#_Toc153647962)

[***RFA group*** 9](#_Toc153647963)

[***No-RFA group*** 9](#_Toc153647964)

[**Figure S1** Bar chart showing the age distribution of the RFA group. 10](#_Toc153647965)

[**Figure S2** Pre-RFA KM cumulative survival curves for VT/VF recurrence by sex. 11](#_Toc153647966)

[**Figure S3** The pre- and post-RFA cumulative survival curves according to clinical presentation. 12](#_Toc153647967)

[**Table S1** Positive genetic testing results in the RFA and No-RFA groups. 13](#_Toc153647968)

[**Table S3** Characteristics of the RFA group according to Sex. 15](#_Toc153647969)

[**Table S4** Characteristics of the RFA group aged ≤30 years and >30 years. 16](#_Toc153647970)

[**Table S5** Characteristics of the RFA group experiencing events according to clinical presentation. 17](#_Toc153647971)

# **SUPPLEMENTARY METHODS**

The study conforms to the principles of the Declaration of Helsinki. The authors guarantee the integrity of the institution data and had approval from a local ethics committee/internal review board. Participants gave informed consent in accordance with the local protocol. None of the study population was included in any previous published registry study.

## ***Study design***

We enrolled symptomatic patients with Brugada Syndrome (BrS) who had received defibrillator implantation for life-threatening symptoms including cardiac arrest or malignant syncope. In all cases, epicardial mapping/ablation under ajmaline administration was proposed as a potential curative measure. However, due to the lack of comprehensive guideline recommendations for epicardial ablation in BrS, we acknowledged the possibility that in our study some eligible patients, according to our inclusion and exclusion criteria, later might be hesitant to undergo this complex and invasive procedure. As a result, initially eligible patients who later opted against RFA ablation would be also included and classified as No-RFA group.

## ***BrS Registry data***

The BrS registry (BrS-R) is an ongoing prospective registry study that initiated in 2015 to assess the role of substrate size as guided by ajmaline administration as risk factor for recurrent VF events as well as the long-term safety and efficacy of epicardial ablation in high-risk symptomatic BrS. Clinical, genetic, electrophysiological, mapping and ablation data, as well as ICD pre- and post-procedure data collection and analysis are all performed at Policlinico San Donato, Clinical Research Institute, Milan, Italy, and the participant’s primary physician is contacted when appropriate. Registry data are only to be used for clinical monitoring and research. Physicians from all over Italy are informed about the BrS-R and are asked to contribute by referring their BrS patients.

Registry data include detailed information on age at diagnosis, prior clinical history, physical examination, 12-lead ECG, echocardiography, diagnostic work-up for syncope, tilting and genetic testing, ICD implantation and interrogation, procedural data, and serial follow-up visits before and after RFA. All other known causes of ST-segment elevation in right precordial leads are excluded before considering the diagnosis of BrS.

The diagnostic work-up for syncope includes: 1) detailed patient’s anamnesis, history of palpitation, pre-syncope (without actual fainting), syncope (with complete loss of consciousness), and/or aborted cardiac arrest (requiring resuscitation maneuvers); 2) detailed family history of syncope, aborted cardiac arrest, or SCD, and age at the event; 3) standard and modified precordial leads (right and left 2nd, 3rd, and 4th intercostal precordial leads); 4) transthoracic 2D-echocardiography; 5) exercise stress testing; 6) 24-hour 12-lead Holter recording; and 7) head-up tilt test to exclude neurally-mediated syncope; 8) coronary angiography in cardiac arrest survivors, and 8) electrophysiological testing to exclude syncope due to supraventricular arrhythmias.

Psychological counseling programs are systematically and uniformly performed before and after the ablation procedure, and throughout the follow-up period, to reduce emotional stress, if necessary. The specific willingness to participate to follow-up visits is critical, since our registry enroll patients from all over Italy, who may be reluctant to systematically undergo follow-up visits far from home, after having undergone epicardial mapping/ablation at IRCCS San Donato Policlinic Hospital.

## ***ICD Implantation and follow-up***

Either a single chamber or dual-chamber intravenous ICD was implanted based on age or patient characteristics. ICD implants were done at San Donato University Hospital in 215 patients at a median of 27 months before enrollment. In all patients, the ICD was programmed to include a single VF zone ≥ 200 bpm/min with one rapid burst of anti-tachycardia pacing (ATP) in the VF zone and up to six shocks at maximum output. In addition to the VF detection zone, a monitoring zone > 180 bpm was included, while the number of beats triggering VT detection was set to 18 beats. Participants were systematically followed up by remote monitoring after ICD implantation and asked to attend follow-up visits at 1, 3, 6, and 12 months during the first year, every 6 months up to 24 months, and included a 12-lead ECG, 24-hour ambulatory ECG monitoring, echocardiography, and ICD interrogation and if necessary, reprogramming.

In the RFA group, the time from ICD implantation to electro-anatomical mapping and RFA was defined as the Pre-RFA follow-up. Events occurring after ICD implant were collected in the registry database. Any ICD intervention, including shock and/or anti-tachycardia pacing (ATP), resulting from polymorphic VT/VF with cycle length within the therapy zone of the device was considered appropriate ICD therapy. The occurrence of three or more separate episodes of sustained VT/VF requiring ICD therapy within a 24-hour period was defined as VF or arrhythmic storm. Absence of VT/VF when a shock was delivered was defined as inappropriate ICD therapy. Non-sustained VT (NSVT) was defined as run of 6 to 30 beats not requiring ICD therapy, and not associated with loss of consciousness.

## ***Genetic testing***

All participants were systematically screened for genetic testing with genomic DNA extracted from peripheral blood and analyzed by NGS technique. The full panel used for Brugada syndrome was composed by *ABCC9, ACTN2, AKAP9, CACNA1C, CACNA2D1, CACNB2, DSG2, GPD1L, HCN4, KCND2, KCND3, KCNE3, KCNE5, KCNE1L, KCNH2, KCNJ8, MOG1, PKP2, SCN1B, SCN2B, SCN3B, SCN5A, SCN10A, SEMA3A, TPM1 and TRPM4*). A subgroup of 8 out of the 206 patients (3.8%) underwent additional genetic testing (Whole Exome Sequencing, encompassing 5000 genes associated with Mendelian conditions) after pre-test genetic counseling due to familial or personal history of genetic conditions different from Brugada syndrome and co-segregating with it. Those conditions were hypertrophic cardiomyopathy (4 patients), homocystinuria (2 patients), long QT syndrome (1 patient) and non-classical congenital adrenal hyperplasia (1 patient). The variants found in those eight patients were in the *MYPBC3, LDB3, CBS, SNTA1* and *CYP21A2* genes (Table S1). Both genetic analyses were performed with a Tru Sight Illumina platform with a medium coverage of 106 X. Gene variants were screened using genomic DNA processed by Next Generation Sequencing (Tru Sight One sequencing kit with Next Seq platform), performed at San Raffaele University-Hospital, Milan, Italy. We used Varsome to obtain an ACMG classification and included all variants with pathogenic (P) or likely pathogenic (LP) classification, but excluded benign (B) or likely benign (LB) and variants of unknown significance (VUS). Other bioinformatics tools we used for variant calling and prediction of pathogenicity were Tru Sight Software Suite, PROVEAN, SIFT, REVEL, GERP, EIGEN, Mutation Taster, FATHMM-MKL and Polyphen. Out of 206 genetically tested RFA patients, 77 were positive (at least one variant in class 3, 4 or 5 identified, Table S1). Among the 77 mutated patients, 47 harbored heterozygous *SCN5A* variant (22.8% of the 206 patients) while 30 patients harbored heterozygous variant in other genes (14.6% of the total 206 RFA patients). The remaining 129 patients (62.6%) were negative (meaning “no variant identified”). Genetic data on the 51 patients who did not undergo epicardial ablation are reported in Table S1.

## ***Abnormal substrate mapping***

We performed substrate mapping by recording and continuously analyzing electrogram (EGM) amplitude and duration in relation to surface QRS waveforms. We used a multipolar catheter to record bipolar [electrograms](https://www.sciencedirect.com/topics/medicine-and-dentistry/electrogram) from RV inflow, anterior free wall, apex, and outflow, and performed epicardial mapping systematically after endocardial mapping at multiple sites. All electrodes were 1-mm in size except for the tip, which was 2 mm, with the smallest interelectrode distance of 2 mm to limit the possibility to consider noise as late activity. We only included acquisitions where the multipolar catheter was stable in each epicardial position, and excluded those with technical issues or catheter-induced extrasystoles, in the setting of a dry epicardium (all liquid was manually drained). EGM morphology, evaluated by expert operators, was considered only if consistent and repetitive for at least 5 consecutive beats, thus avoiding artifacts. The 12-lead surface ECG was continuously recorded throughout the procedure.

We used a three-dimensional mapping system (CARTO 3, Biosense Webster, Diamond Bar, CA, USA), to obtain electroanatomical maps at baseline and after administration of ajmaline (up to 1 mg/Kg in 5 minutes). A steerable sheath (Agilis St Jude Medical, St Paul, MN, USA) facilitated manipulations of the epicardial RV mapping catheter. EGMs were detected only by the electrode pairs of a dedicated decapolar catheter (DECANAV catheter, Biosense Webster, Diamond Bar, California, USA). This procedure provided high-density, color-coded endocardial and epicardial electroanatomical maps (EAM) simultaneously. We created potential duration maps (PDM) by collecting the duration of each EGM, resulting in color-coded electroanatomical maps showing the regions displaying the shortest (<110 ms, red color) and the longest (>150 ms, purple color) durations. We identified arrhythmogenic substrate areas and systematically validated them using CARTO 3 system (Biosense Webster). We filtered bipolar EGMs from 16 to 500 Hz with 0.32 or 0.39 mV gain and displayed them at speed of 200 mm/s. We considered electrograms without delayed components exhibiting amplitudes smaller than 0.5 mV as low voltage. The abnormal substrate area was determined at baseline, and during ajmaline challenge (up to 1 mg/kg in 5 min) with complete substrate size considered as the substrate area (cm^2^) elicited by ajmaline challenge. We quantified the substrate area only on the epicardial aspect since abnormal potentials were never found at the endocardial site correspondent to the affected epicardial area. Before ajmaline challenge, we commonly observed minor electrical abnormalities in the right ventricle (RV) outflow tract (RVOT), but not in the rest of the RV. After ajmaline challenge, the abnormalities usually extended from RVOT epicardium to include the RV anterior wall. After determining the substrate size, we used a stimulation protocol to assess the VT/VF induction**,** including up to 3 extrastimuli from 2 sites (either apex or RVOT) using 10-ms decrements, down to the refractory period or to a minimum of 180 ms. If the patient was not inducible at baseline, we administered ajmaline again (up to 1 mg/kg) to verify inducibility during drug challenge.

## ***Substrate ablation***

Radiofrequency ablation was systematically performed during sinus rhythm at the epicardial sites where ajmaline infusion had defined abnormal areas. The RFA procedure was continued until all electrogram abnormalities on the epicardial electroanatomic maps were eliminated, and until the type-1 BrS pattern was no longer inducible, even after an ajmaline challenge. The ablation procedure began with the region that had the longest activity and then progressed gradually to the areas with less delayed and fragmented potentials. After completion of ablation, we remapped the substrate area and systematically reinfused ajmaline to ensure elimination of all abnormal potentials elicited by ajmaline, while verifying elimination of the type 1 BrS pattern on 12-lead ECG. If the type-1 BrS pattern reappeared during ajmaline infusion, we recorded a new epicardial map to locate the source of the remaining abnormal signals and performed another substrate ablation to finally normalize the PDM and the ECG pattern. We did not evaluate VT/VF induction until the final ajmaline challenge demonstrated both the abolition of abnormal epicardial activity and elimination of the BrS-ECG pattern. Reduction of voltage to < 0.5 mV (dense scar tissue) with disappearance of all components of fractionated potentials before and after ajmaline injection indicated complete abolition of the arrhythmogenic substrate.

# **SUPPLEMENTARY RESULTS**

## **RFA Group (206 patients)**

Age distribution of the RFA group is shown in the Figure S1. Notably, none of these 206 patients experienced severe complications or lasting effects following ACA or syncope presentation. Patients with ACA had uneventful clinical histories, with recognized triggers including concurrent febrile illness in 4 patients, ethanol consumption in 6 patients, and stressful conditions in 5 patients. Similarly, triggers for syncope included fever (7 patients), ethanol consumption (8 patients), emotional stress (5 patients) and extremely high temperature in crowded settings (4 patients).

Runs of polymorphic VT/VF were observed in the intensive care unit in 10 patients immediately after syncope or later on, prior to ICD implantation, in 42 additional patients who experienced palpitations during Holter monitoring. Coronary angiography yielded negative results in cardiac arrest survivors while tilt testing was negative in patients with syncope suspected to be related to VT/VF after undergoing a comprehensive diagnostic workup. A total of 79 patients (including 48 with ACA) who had inducible VF and occasional palpitations or dizziness were prescribed chronic quinidine therapy, with dosages adjusted up to 900 mg per day, prior to ICD implantation. However, due to intolerable side effects, 53 patients discontinued the drug within a few weeks. ACA patients displayed higher rates of VF inducibility compared to syncope patients, while no significant differences were noted within the control group between ACA and syncope patients (Table S2). Despite the RFA group being predominantly male, no significant differences in baseline characteristic were observed based on sex (Table S3). The RFA group included 52 patients aged 30 years or younger, and no significant differences in baseline characteristics were noted observed between these two subgroups (Table S4). Baseline type 1 ECG pattern ≥ 2mV was observed in a minority of study population. Among patients with syncope, 35 showed borderline coved-type ECG patterns (less than 0.2 mV) and thus were considered as non-type 1 ECG pattern, according to guideline recommendations.

## **No-RFA group (51 patients)**

Among a total of 51 patients declining ablation, 16 experienced ACA and 35 had malignant syncope. Cardiac arrest survivors received immediate cardiopulmonary resuscitation and shocks for documented VF. All of these cases displayed negative results in coronary angiography and exhibited uncomplicated neurological recoveries. Among the 35 patients experiencing “malignant” syncope, only 5 of these patients had documented self-terminating VT/VF episodes. Syncope occurred at rest without prodromal signs in 34 patients, and in one patient, it occurred during a febrile episode. Tilt testing yielded negative results in all cases without arrhythmia documentation. Before their ICD implantation, 29 patients with inducible sustained VF were put on chronic quinidine therapy, with doses adjusted up to 900 mg per day. However, due to intolerable side effects, 19 of these patients stopped the therapy a few months later. The remaining patients continued quinidine therapy after ICD implantation.

Among patients with syncope, 8 showed borderline coved-type ECG patterns less than 0.2 mV.

## **ICD complications**

### ***RFA group***

During follow-up*,* 20 patients (9.7%) experienced inappropriate shocks. Of these, 15 patients had multiple inappropriate shocks due to paroxysmal atrial fibrillation with a fast ventricular rate, while 5 patients had a single inappropriate shock due to exercise-related sinus tachycardia. Additionally, 30 patients (14.6%) had device-related complications, with lead dislocation in 16 patients and generator migration in 14 patients, leading to revision of the device in all cases.

### ***No-RFA group***

During follow-up***,*** 12 patients (23.5%) experienced multiple (7 patients) or single (5 patients) inappropriate shocks. Multiple inappropriate shocks were due to paroxysmal atrial fibrillation with a fast ventricular rate while a single inappropriate shock was due to sinus tachycardia related to exercise. Additionally, 9 patients had device-related complications, including lead dislocation in 7 patients and electrode fracture in 2 patients, requiring extraction and replacement.

# **Figure S1** Bar chart showing the age distribution of the RFA group.

**
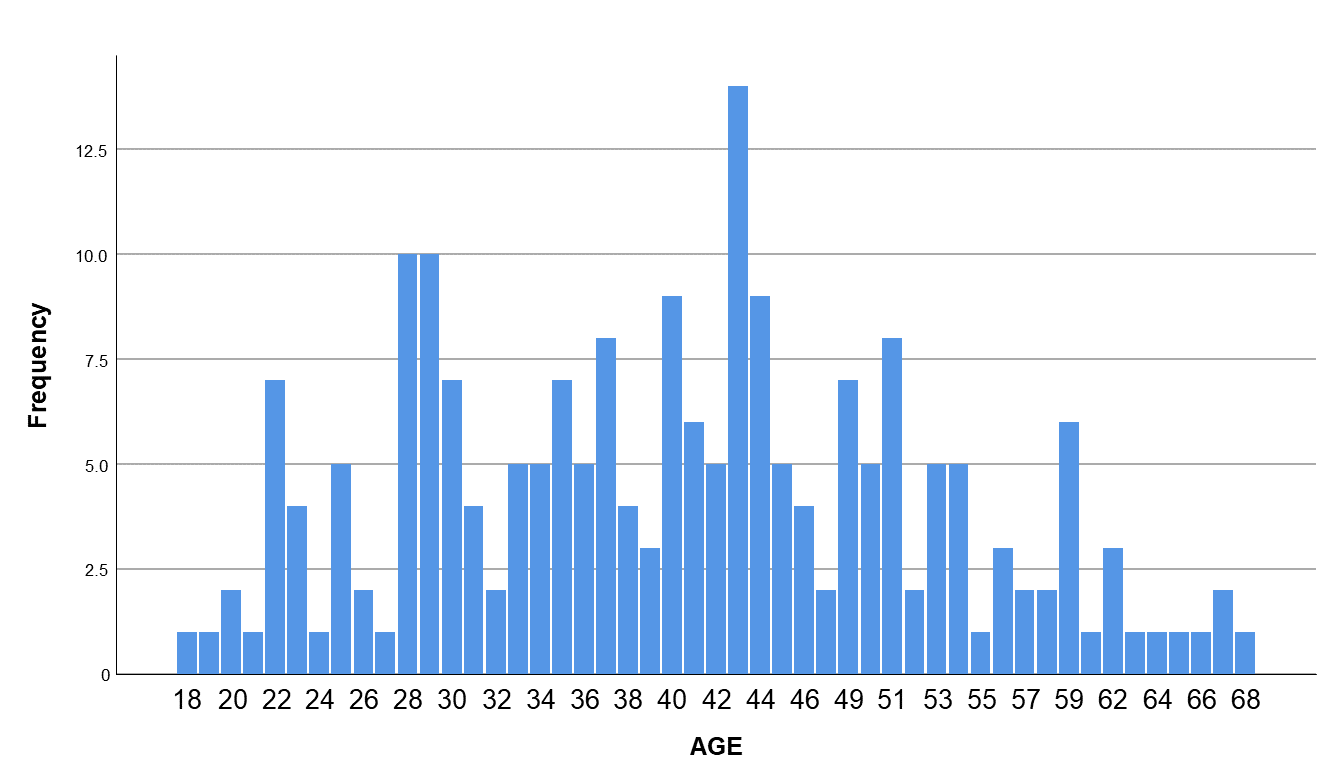
**

# **Figure S2** Pre-RFA KM cumulative survival curves for VT/VF recurrence by sex.

**
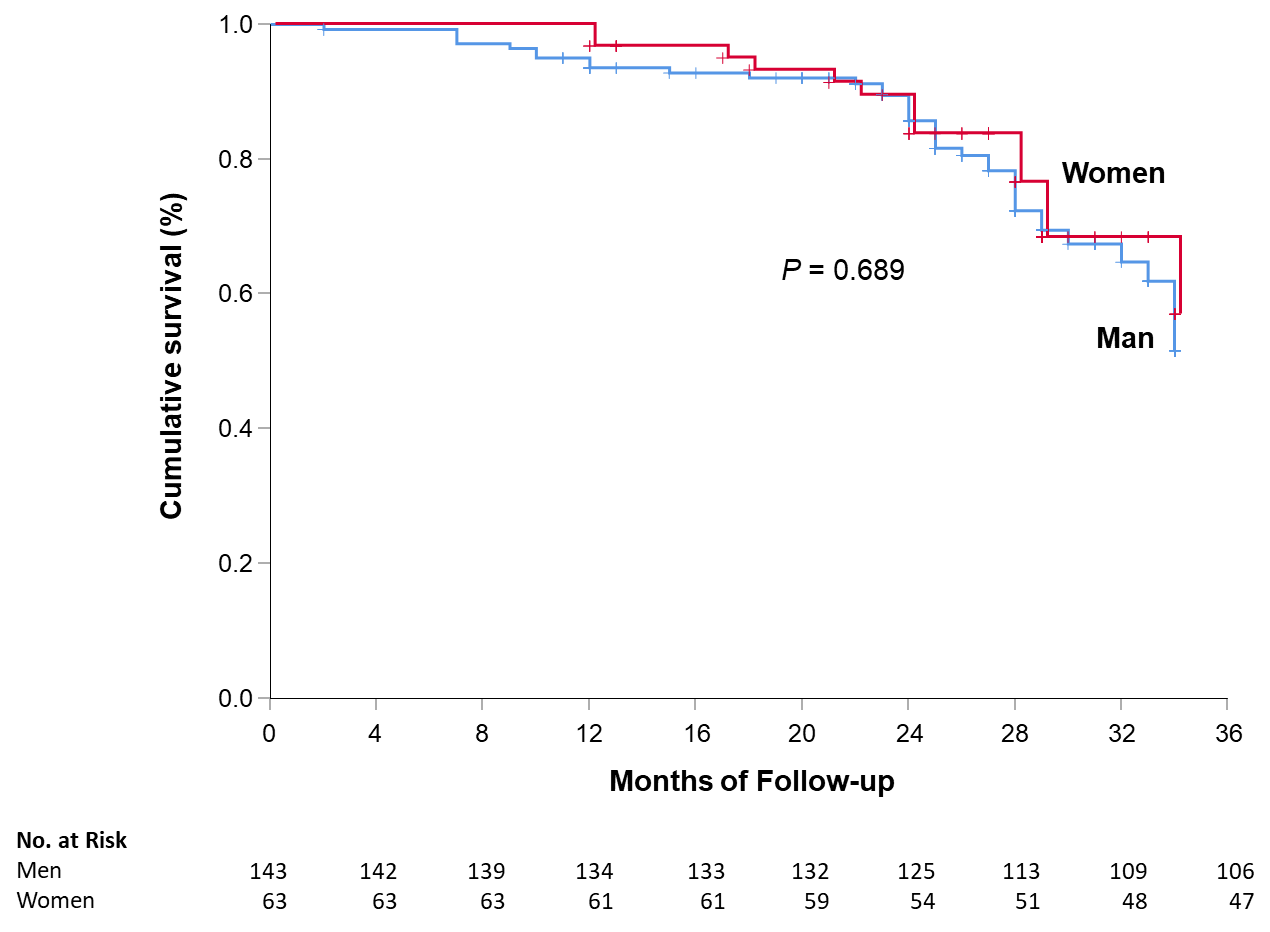
**

# **Figure S3** The pre- and post-RFA cumulative survival curves according to clinical presentation.

In figure S3A the time of ICD implant was the baseline, and the time to first event the outcome variable. In figure S3B the time of RFA ablation was the starting point, and time to the first event the outcome variable.


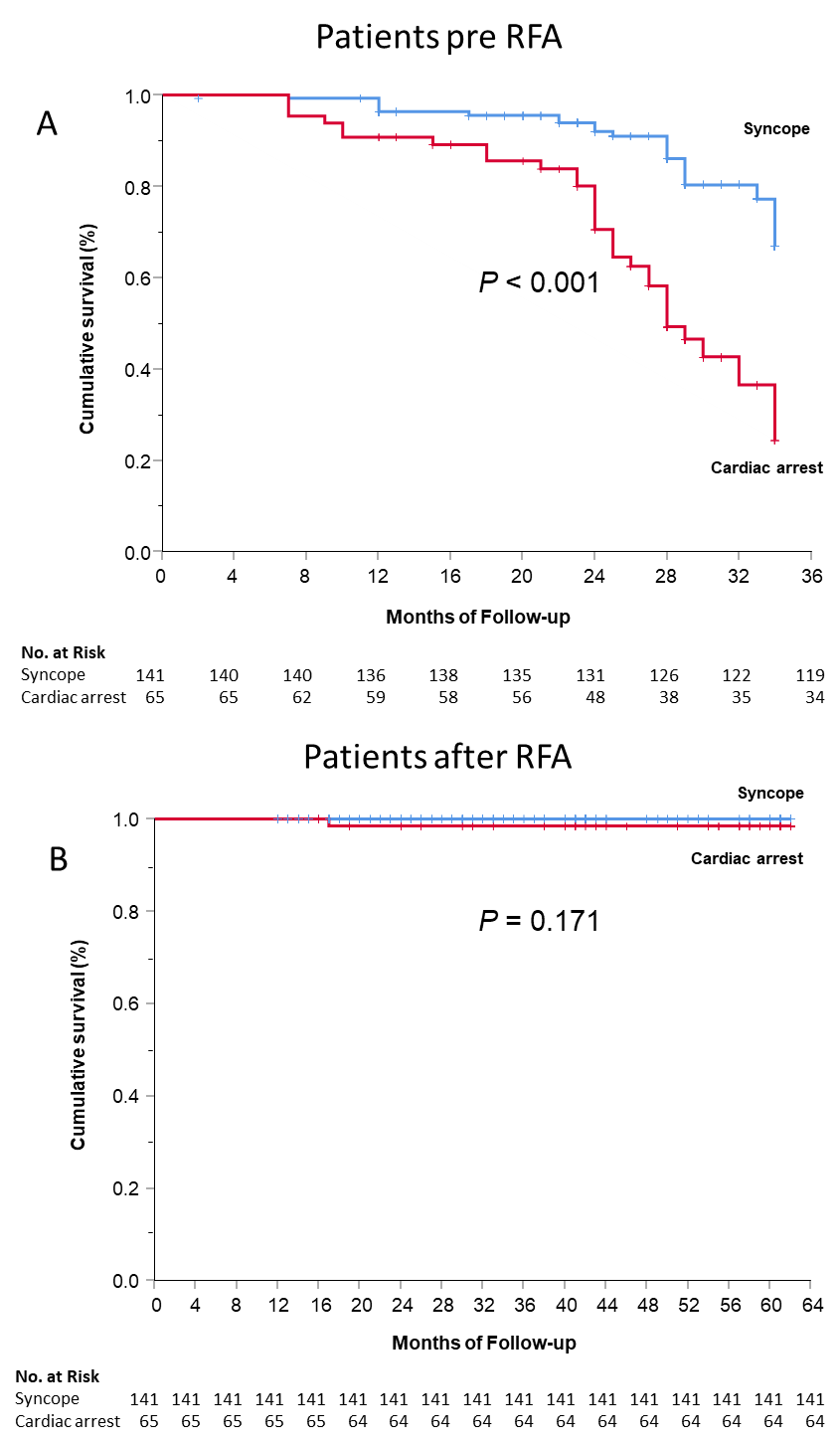


# **Table S1** Positive genetic testing results in the RFA and No-RFA groups.

| ***SCN5A gene* variant** | **Frequency** |  | ***No SCN5A* gene variant** | **Frequency** |
| --- | --- | --- | --- | --- |
| **RFA GROUP** | | | | |
| SCN5A: c.1041C>A | 2 |  | ABCC9: c.2851G>A | 1 |
| SCN5A: c.1045G>A | 1 |  | ACTN2: c.2147C>T | 1 |
| SCN5A: c.1140+2T>C | 1 |  | AKAP9: c.611A>G | 1 |
| SCN5A: c.1890G>A | 1 |  | CACNA2D1: c.2645T>C | 1 |
| SCN5A: c.2039G>A | 1 |  | CACNB2: c.1180G>A | 2 |
| SCN5A: c.2077C>T | 1 |  | CBS: c.129G>A (heteroz) | 1 |
| SCN5A: c.2182G>A | 1 |  | CBS: c.129G>A (homoz) | 1 |
| SCN5A: c.2236G>A | 1 |  | CYP21A2: c.1022G>C | 1 |
| SCN5A: c.2314G>A | 1 |  | DSG2: c.1173C>A | 1 |
| SCN5A: c.2414C>T | 1 |  | DSG2: c.3046G>T | 1 |
| SCN5A: c.2441G>A | 3 |  | KCND3: c.1531A>G | 1 |
| SCN5A: c.2542A>G | 1 |  | KCNE3: c.157C>T | 2 |
| SCN5A: c.3214_3215delins | 1 |  | KCNE3: c.241C>T | 1 |
| SCN5A: c.3673G>A | 1 |  | KCNH2: c.3457C>T | 1 |
| SCN5A: c.3840+1G>A | 1 |  | LDB3: c.91C>G | 1 |
| SCN5A: c.3917G>A | 4 |  | MYBPC3: c.1828G>C | 1 |
| SCN5A: c.3946C>T | 2 |  | MYBPC3: c.3335_3337dup | 1 |
| SCN5A: c.4057G>A | 1 |  | MYBPC3: c.913_914del | 1 |
| SCN5A: c.4140C>G | 1 |  | PKP2: c.2200A>G | 1 |
| SCN5A: c.422T>A | 1 |  | SCN10A: c.1192A>G | 1 |
| SCN5A: c.4437+5G>A | 1 |  | SCN10A: c.800T>A | 1 |
| SCN5A: c.4516C>A | 1 |  | SCN1B: c.134G>A | 1 |
| SCN5A: c.4534C>T | 1 |  | SCN3B: c.260C>G | 1 |
| SCN5A: c.4536_4537delGCi | 1 |  | SNTA1: c.1256G>A | 1 |
| SCN5A: c.4700_4701del | 2 |  | TPM1: c.571C>T | 1 |
| SCN5A: c.4813G>T | 1 |  | TRPM4: c.2264G>T | 1 |
| SCN5A: c.481G>A | 2 |  | TRPM4: c.2309G>T | 1 |
| SCN5A: c.4850_4852delTCT | 1 |  | TRPM4: c.2981C>T | 1 |
| SCN5A: c.4867C>T | 3 |  |  |  |
| SCN5A: c.655C>T | 2 |  |  |  |
| SCN5A: c.665G>A | 1 |  |  |  |
| SCN5A: c.733C>A | 1 |  |  |  |
| SCN5A: c.809_812dup | 2 |  |  |  |
| SCN5A: c.880G>A | 1 |  |  |  |
| **No-RFA group** | | | | |
| SCN5A: c.1030G>T | 1 |  | LMNA; c.606A>G | 1 |
| SCN5A: c.1045G>A | 1 |  |  |  |
| SCN5A: c.127C>T | 1 |  |  |  |
| SCN5A: c.1657G>T | 1 |  |  |  |
| SCN5A: c.2091G>A | 2 |  |  |  |
| SCN5A: c.3697A>T | 1 |  |  |  |
| SCN5A: c.3917G>A | 2 |  |  |  |
| SCN5A: c.4057G>A | 1 |  |  |  |
| SCN5A: c.4285G>A | 1 |  |  |  |
| SCN5A: c.5129C>T | 1 |  |  |  |

**Table S2** Comparison of characteristics between the RFA and No-RFA groups, according to their clinical presentation.

| **Characteristics** | **RFA-GROUP** | |  | **No-RFA GROUP** | |  |  |  |
| --- | --- | --- | --- | --- | --- | --- | --- | --- |
|  | **Cardiac Arrest^a^ (n=65)** | **Syncope^b^**  **(n=141)** |  | **Cardiac Arrest^c^ (n=16)** | **Syncope^d^**  **(n=35)** |  | ***P-value***  **(a vs c)** | ***P-value***  **(b vs d)** |
| Male sex − n (%) | 48 (73.8) | 95 (67.4) |  | 10 (62.7) | 24 (68.6) |  | 0.349 | 0.670 |
| Age at diagnosis − Years, mean ± SD  Min-Max range | 41.2 ± 10.7  22-63 | 40.1 ± 12.1  18-68 |  | 37.4 ± 10.2  18-53 | 39.9 ± 7.3  19-55 |  | 0.545 | 0.334 |
| Pre ICD type 1 ECG pattern − n (%) | 19 (29.2) | 24 (17.0) |  | 2 (12.5) | 9 (25.7) |  | 0.045 | 0.466 |
| Family history of SCD − n (%) | 16 (24.6) | 55 (39.0) |  | 7 (43.8) | 11 (31.4) |  | 0.043 | 0.393 |
| Positive *SCN5A* variant − n (%) | 17 (26.2) | 30 (21.3) |  | 4 (25.0) | 8 (22.9) |  | 0.438 | 1.000 |
| Inducible VT/VF | 59 (90.8) | 55 (39.0) |  | 9 (56.3) | 20 (57.1) |  | < 0.001 | 0.952 |
| Substrate size − cm^2^  Min-Max range | 11.0 (6.4-14.1)  0-56.6 | 4.2 (0.9-10.0)  0-23.9 |  | - | - |  | < 0.001 | - |
| Substrate size after ajmaline − cm^2^  Min-Max range | 25.0 (19.3-28.2)  12.7-64.2 | 14.2 (11.7-17.6)  2.3-36.6 |  | - | - |  | < 0.001 | - |
| *ICD follow-up − months  Min-Max range | 25 (19-29)  7-34 | 28 (23-29)  2-34 |  | 25 (22-27)  3-33 | 27 (24-30)  12-34 |  | 0.052 | 0.064 |
| **Outcome follow-up − months  Min-Max range | 58 (41-61)  16-62 | 33 (24-43)  12-62 |  | 33 (24-40)  8-62 | 34 (25-48)  9-62 |  | < 0.001 | 0.633 |

Data are medians and interquartile ranges except when indicated.

* For the RFA group, 'ICD follow-up' refers to the period between ICD implantation and RFA ablation. In the control group, 'ICD follow-up' denotes the time span between ICD implantation and the decision to refuse ablation.

** For the RFA group, 'outcome follow-up' refers to the time span between RFA ablation and the most recent follow-up contact. In the No-RFA group, 'outcome follow-up' represents the period from when the ablation was declined to the latest follow-up contact. This ensures a comparable starting point for both groups by excluding the interval between ICD implantation and the decision to decline the procedure.

Abbreviations: IQR: Interquartile range; BrS: Brugada Syndrome; SCD: Sudden cardiac death; VT/VF: Ventricular tachycardia/fibrillation RFA: Radiofrequency ablation.

**Table S3** Characteristics of the RFA group according to Sex.

| **Characteristic** | **Sex** | | ***P-value*** |
| --- | --- | --- | --- |
|  | **Men**  **(n=143)** | **Women**  **(n=63)** |  |
| Age at diagnosis – years  Min-Max | 40.3 ± 10.9  18-68 | 40.9 ± 13.2  20-67 | 0.764 |
| Spontaneous Type 1 ECG pattern − n (%) | 35 (24.5) | 8 (12.7) | 0.055 |
| Family history of SCD − n (%) | 46 (32.2) | 25 (39.7) | 0.296 |
| Positive SCN5A − n (%) | 31 (21.7) | 16 (25.4) | 0.558 |
| Inducible VT/VF − n (%) | 80 (55.9) | 34 (54.0) | 0.793 |
| Aborted cardiac arrest − no. (%) | 48 (33.6) | 17 (27.0) | 0.349 |
| Appropriate ICD therapy | 37 (35.9) | 16 (25.4) | 0.942 |
| Median ICD shocks (IQR)  Min-Max | 2 (1-2)  1-10 | 1 (1-2)  1-3 | 0.244 |
| Median pre RFA follow-up (IQR) – months  Min-Max | 27 (22-29)  2-34 | 29 (23-29)  12-34 | 0.328 |
| Median baseline substrate size (IQR) − cm^2^  Min-Max | 6.0 (1.7-11.5)  0-56.6 | 5.5 (2.5-12.2)  0-23.9 | 0.835 |
| Median substrate size after Ajmaline (IQR) − cm^2^  Min-Max | 16.9 (13.0-25.1)  2.3-64.2 | 16.2 (11.8-21.5)  4.2-35.9 | 0.155 |

IQR denotes interquartile range.

**Table S4** Characteristics of the RFA group aged ≤30 years and >30 years.

| **Characteristic** | **Age groups** | | ***P-value*** |
| --- | --- | --- | --- |
|  | **≤30 years**  **(n=52)** | **>30 years**  **(n=154)** |  |
| Male sex − n (%) | 34 (65.4) | 109 (70.8) | 0.465 |
| Spontaneous Type 1 ECG pattern − n (%) | 10 (19.2) | 33 (21.4) | 0.736 |
| Family history of SCD − n (%) | 22 (42.3) | 49 (31.8) | 0.169 |
| Positive SCN5A − n (%) | 13 (25.0) | 34 (22.1) | 0.664 |
| Inducible VT/VF − n (%) | 27 (51.9) | 87 (56.5) | 0.567 |
| Prior cardiac arrest − no. (%) | 13 (25.0) | 52 (33.8) | 0.240 |
| ICD therapy | 11 (21.2) | 42 (27.3) | 0.383 |
| Median pre RFA follow-up (IQR) − months  Min-Max | 25.5 (24.0-29.0)  7-34 | 28.0 (21.0-29.0)  2-34 | 0.933 |
| Median baseline substrate size (IQR) − cm^2^  Min-Max | 5.3 (1.6-10.7)  0.1-56.6 | 6.2 (2.1-12.0)  0-36.6 | 0.429 |
| Median substrate size after Ajmaline (IQR) − cm^2^  Min-Max | 16.4 (13.0-24.8)  6.4-64.2 | 16.7 (12.2-24.5)  2.3-44.2 | 0.988 |

IQR denotes interquartile range.

# **Table S5** Characteristics of the RFA group experiencing events according to clinical presentation.

| **Characteristic** | **Arrhythmic events** | | ***P-value*** |
| --- | --- | --- | --- |
|  | **Aborted Cardiac Arrest**  **(n=31)** | **Syncope**  **(n=22)** |  |
| Male sex − no. (%) | 25 (80.6) | 12 (54.5) | 0.041 |
| Age at diagnosis − years  Min-Max | 41.2±11.2  22-63 | 41.2±10.7  20-59 | 0.950 |
| Spontaneous Type 1ECG pattern − n (%) | 10 (32.3) | 7 (31.8) | 0.975 |
| Family history of SCD − n (%) | 3 (9.7) | 9 (40.9) | 0.017 |
| Positive SCN5A − n (%) | 14 (45.2) | 19 (86.4) | 0.002 |
| Inducible VT/VF − no. (%) | 27 (87.1) | 12 (54.5) | 0.008 |
| More than 1 Appropriate Shock − no. (%) | 22 (71.0) | 5 (22.7) | <0.001 |
| Median baseline substrate size (IQR) − cm^2^  Min-Max | 14.0 (12.1-15.6)  9.0-56.6 | 13.1 (11.1-14.5)  9.2-23.9 | 0.098 |
| Median substrate size after Ajmaline (IQR) − cm^2^  Min-Max | 26.8 (22.1-28.9)  18.2-64.2 | 15.1 (11.6-23.3)  6.4-36.6 | < 0.001 |

Plus–minus values are means ±SD. IQR denotes interquartile range.
